# Spontaneous healing of a ruptured anterior cruciate ligament: a case series and literature review

**DOI:** 10.1186/s40634-022-00566-9

**Published:** 2023-02-04

**Authors:** Leonardo Previ, Edoardo Monaco, Alessandro Carrozzo, Gianluca Fedeli, Alessandro Annibaldi, Matteo Romano Cantagalli, Giorgio Rossi, Andrea Ferretti

**Affiliations:** AOU Sant’Andrea, La Sapienza University of Rome, Rome, Italy The Investigations Were Performed at AOU Sant’Andrea, La Sapienza University of Rome, Rome, Italy

**Keywords:** Anterior cruciate ligament, ACL, Healing, Spontaneous healing

## Abstract

**Purpose:**

The anterior cruciate ligament is probably one of the ligaments with the lowest healing potential. Many authors have reported cases of spontaneous healing but nowadays it is difficult to predict successful healing of an anterior cruciate ligament rupture and, even more, residual functionality and capability to return to sport. The aim of this study was to investigate cases of spontaneous healing in a population that received non-surgical treatment after anterior cruciate ligament rupture and to perform an updated review of contemporary literature.

**Methods:**

The authors retrospectively reviewed patients who suffered from an acute complete anterior cruciate ligament rupture and underwent non-surgical treatment. No specific rehabilitation protocol was prescribed. A new magnetic resonance imaging study was conducted 6 months after the injury for all patients. A literature review was conducted regarding spontaneous healing of the anterior cruciate ligament. The papers included in the analysis were reports of any level of evidence, written in English, Italian, or French languages; articles were excluded if they reported non-human studies, histological studies, studies conducted without magnetic resonance imaging or arthroscopic second look, or partial anterior cruciate ligament tear.

**Results:**

Case series: Six patients were enrolled in the study. All patients had a proximal anterior cruciate ligament lesion. The minimum follow-up was 13 months (range 6–20 months). At the last follow-up the mean score on the Lysholm scale was 97, the mean IKDC score was 94, and the mean KOOS score was 96. All patients returned to their own sport activities; no one reported significant differences. The magnetic resonance imaging study at 6 months revealed an end-to-end continuous anterior cruciate ligament with homogeneous signal. No one had any new knee injury at last follow-up. Literature review: A search of comprehensive databases retrieved 1057 articles; 8 full-text articles met the eligibility criteria. The studies were heterogeneous regarding the populations analysed, sport activity level, treatment applied, healing definition, and follow-up. The failure rate of non-surgical management ranged among the papers from 0 to 73%.

**Conclusions:**

The study findings show that spontaneous anterior cruciate ligament healing is possible and there are chances of clinical recovery for patients not suitable for surgery. However, there is still a lack of evidence about predictors, clinical outcomes, and adequate rehabilitation protocols.

## Introduction

The spontaneous healing capability of an injured anterior cruciate ligament (ACL) has been investigated using tissue culture [1], electron microscopy [2], and the reverse transcription-polymerase chain reaction [3]. Various factors that adversely affect the healing of an injured ACL are reported in the literature. Fibrin–platelet clot formation, which is necessary to initiate tissue healing, is inhibited by surrounding synovial fluid [4, 5]. Also, quadriceps activation induces an anterior drawer movement that can pull the stumps apart and may result in a lengthened ligament [6]. The ligament stumps’ position is influenced by gravity force and there may be no contact between them [7]. Another argument in favour of the traditional consideration of the ACL as a ligament with scarce healing capacity is the poor clinical outcomes of a direct repair [8, 9].

The mainstay of ACL rupture treatment is actually considered to be surgery [10]. In the literature, however, there are a few reports of patients that obtain an improvement in knee stability with spontaneous ACL healing without surgical management [7, 10–17].

Recent histological and magnetic resonance imaging (MRI) studies have suggested that tears in the proximal one-third of the ACL, the region with greatest perfusion from the middle geniculate artery, may have an intrinsic healing response similar to that of the medial collateral ligament (MCL) [18, 19].

The primary aim of this study was to investigate cases of spontaneous healing in a population that received non-surgical treatment after ACL rupture. Another objective was to perform an updated literature review to report the outcomes, evidence on indications, and conservative treatment strategies for spontaneous ACL healing in contemporary literature.

We hypothesized that in a small proportion of patients that do not undergo surgical management of an ACL injury, a clinical recovery obtaining a stable knee may be possible.

## Methods

### Case series

A retrospective analysis was conducted on patients who received non-surgical treatment for ACL injury in the emergency department of our institution (Azienda Ospedaliera Universitaria Sant’Andrea, La Sapienza University of Rome) from January 1, 2015 to April 30, 2020. Patients were evaluated by the senior authors (ED, AC) and the diagnosis was based on clinical examination (Lachman and pivot shift tests) and confirmed by MRI. MRIs were performed with a 1.5 T MRI scanner (Symphony; Siemens Medical Solutions). All patients underwent both T1-weighted and T2-weighted imaging, performed in the axial, sagittal, and coronal planes, in sections with 3 to 4 mm thickness.

Patients were not assigned to acute ACL surgery if they met one or more of the following criteria: very low demand patients (Tegner ≤ 2), inability to undergo surgery within 15 days of injury, had an ACL injury during the first 2 months of the COVID-19 pandemic – March and April 2020 – that did not require urgent treatment (e.g., bucket-handle meniscal lesion), Kellgren Lawrence grade III or IV change in any compartment, or refusal to receive surgical management. The patients who did not undergo acute ACL surgery were referred for periodic clinical evaluation to determine whether or not surgery was needed at a later date.

### Rehabilitation

The initial management included an extension brace and walking with crutches (partial weight bearing) for 3 weeks. The focus was on activation of the vastus medialis oblique and isometric quadriceps exercises. Subsequently, patients were followed up by a physiotherapist at least for 2 months to regain the full range of motion (ROM) and improve strength and proprioception of the injured lower limb.

### Follow-up

Clinical evaluations were performed monthly for the first 3 months. Then, an MRI was conducted 6 months after the injury. At the last follow-up, patients were evaluated according to the Lysholm scale [20], the International Knee Documentation Committee (IKDC) score, and Knee injury and Osteoarthritis Outcome Score (KOOS) [21]. The physical examination comprised a Lachman test, pivot shift test, and measurement of the side-to-side difference in millimetres between injured and healthy knees with a KT-1000™ arthrometer (MEDmetric Corp, San Diego, CA, USA). A retrospective assessment of the MRIs was performed to classify the ACL injury site according to Van der list et al. [22]: type I tear (proximal avulsion tear, located at > 90% of distal–proximal length), type II tear (proximal tear, located at 75%–90% of distal–proximal length), type III tear (mid-substance tear, located at 25%–75% of distal–proximal length), type IV tear (distal tear, located at 10%–25% of distal–proximal length), or type V tear (distal avulsion tear, located at < 10% of distal–proximal length).

Patients were defined as healed based on clinical and MRI criteria: no giving-way episodes reported after the injury, negative Lachman and jerk tests, and a continuous ACL with a Howell grade 1 or 2 at MRIs.

### Literature review: search strategy and eligibility criteria

A review was conducted on contemporary literature regarding spontaneous ACL healing. The Boolean search was performed on May 1, 2022, using the following keywords and operators: ((ACL) OR (Anterior cruciate ligament)) AND ((Spontaneous) OR (autonomous)) AND (Healing). The databases investigated were PubMed, Scopus, and the Cochrane Library. The Preferred Reporting Items for Systematic Reviews and Meta-analysis (PRISMA) guidelines were used [23].

Firstly, articles were screened by title and abstract. The screening was conducted separately by 2 independent observers (L.P. and G.F.). To identify included studies, the PICOS criteria were used [24]. Studies were considered eligible to be included in the review if they included patients diagnosed with a complete ACL rupture (P) and who had non-surgical treatment (I). A control group (C) was not considered an inclusion criterion, and ACL healing assessed using MRI or with an arthroscopic second look was considered the outcome measure (O). There were no restrictions on study type (S). Clinical reports of any level of evidence, written in the English, French, or Italian languages, were included for further analyses. Exclusion criteria were articles written in languages other than English, French, or Italian; non-human studies; histological studies; studies conducted without assessing ACL healing by MRI or arthroscopic second look; studies including patients who suffered from partial ACL tear; white papers; abstracts.

Then, full texts of selected articles were screened, with further exclusions according to the previously described criteria. Reference lists from the selected papers were also screened.

Two independent investigators extracted data and in case of discrepancies the final decision was made by a third investigator (E.M.). Relevant data (study design, year of publication, number of patients, age of patients, type of lesion, associated lesions, rehabilitation type, activity level, failure rate, follow-up duration, KT-1000, IKDC, Lysholm score) were then extracted and collected in a unique database (Table 1). Two independent authors performed the screening process, study analysis, and data tabulation separately. A final summary was obtained by consensus, with discrepancies discussed with a third reviewer (L.P., G.F., A.C.).

**Table 1:** Demographic data

No	Age	Sex	Side	BMI	Mechanism of injury	Type of lesion	Associated lesion
1	42	F	L	24.2	Dance	Type I	MCL grade II
2	49	M	R	26.3	Soccer	Type I	None
3	35	F	L	23.1	Volleyball	Type II	None
4	36	M	L	24.0	Tennis	Type I	None
5	25	F	L	22.3	Dance	Type II	None
6	59	F	R	27.8	Tennis	Type I	MCL grade II

*Abbreviations*: *F*, female, *M*, male, *MCL*, medial collateral ligament

### Statistical analysis

Calculations were made using Microsoft Excel for MacOS (Version 16.58; Microsoft Corporation, WA, USA). Descriptive data analysis was conducted depending on the nature of the considered criteria. For quantitative data, this included the number of observed (and missing, if any) values and the mean, SD, and range. For qualitative data, this included the number of observed (and missing, if any) values and the number and percentage of patients per group. For the literature review, data were extracted from included studies to determine the number of cases of spontaneous healing of ACL and the total number of patients in each group. Results are presented using means and ranges or proportions.

## Results

### Case series

**Fig. 1:**
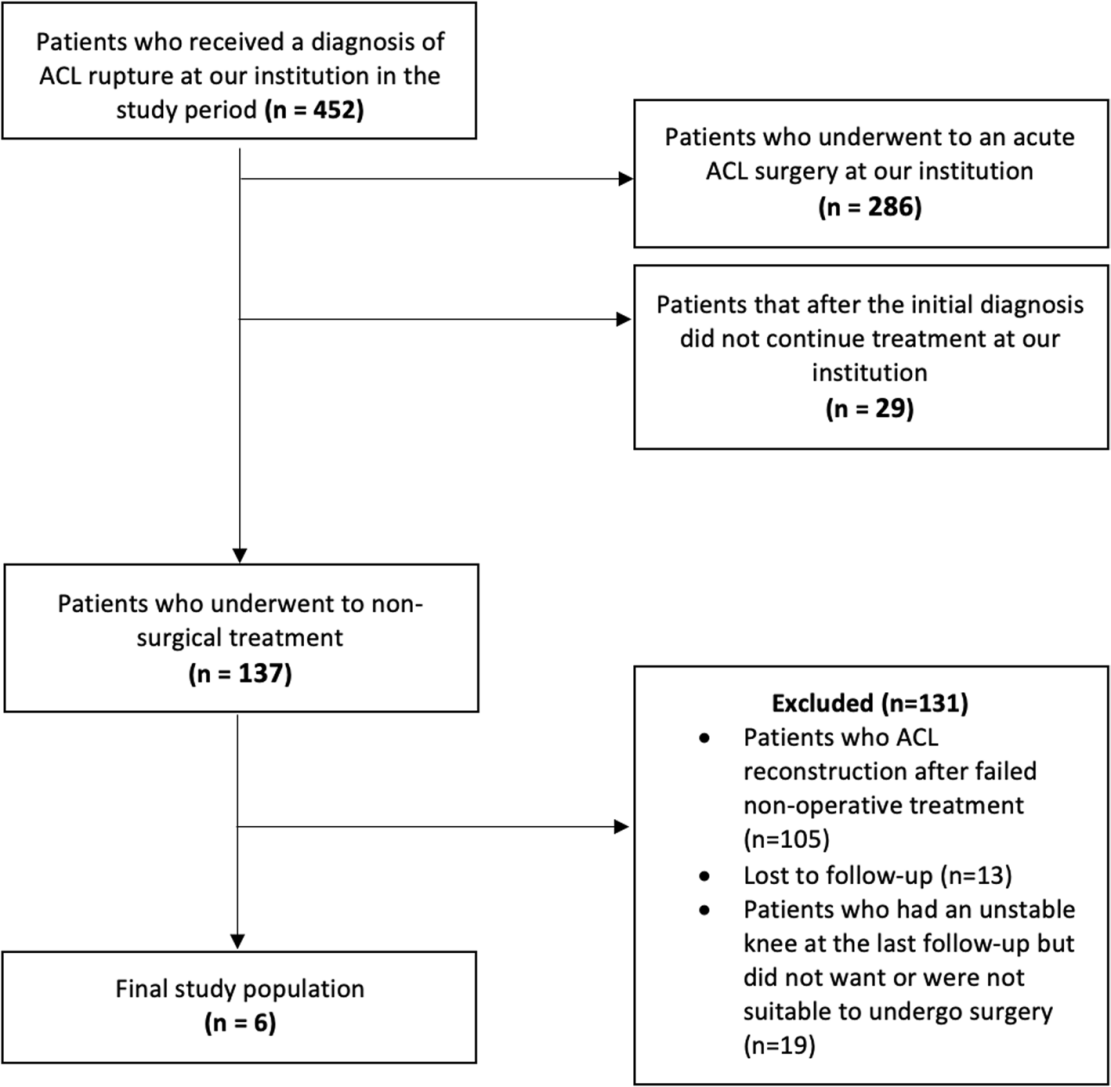
The study flow chart in line with the STROBE (Strengthening the Reporting of Observational Studies in Epidemiology) statement (http://www.strobestatement.org). ACL, anterior cruciate ligament

The final population of the case series was composed of 6 patients and the study flow is presented in Fig. 1. All the patients received a diagnosis of complete ACL rupture by clinical and MRI assessment within 10 days of knee trauma. Mean age was 34.3 years (range 25–59 years); there were 4 females and 2 males. Two patients had a right ACL injury and 4 a left injury. According to Van der list et al. [25] radiographic classification of location of injury, 4 patients sustained a type I ACL lesion, and 3 patients had a type II injury. Demographic data for the 6 patients are represented in Table 1.

All patients were recreational athletes, used to practise sport at least once a week, and suffered the injury during sport activity. Two of them practised dance, two played tennis, one played volleyball, and one played football.

The mean follow-up was 13 months (range 6–20); at last clinical evaluation, 4 patients had a grade 0 Lachman test and 2 a grade I; 3 patients had no pivot shift sign, and 3 patients had a pivot shift graded as glide. The average KT-1000 arthrometer manual side-to-side maximum value was 2.6 mm (range 1–6 mm). The mean KOOS was 96 (range 94–98); the average Lysholm score was 97 (range 90–100); the mean subjective IKDC score was 94 (range 89–98), 4 patients being graded as ‘A’ and 2 as ‘B’ at objective IKDC evaluation. Clinical results are displayed in Table 2. All patients returned to pre-injury sport activity level except 1 who reported decreased sport activity due to the fear of re-injury.

**Table 2:** Results and scores 6 months at the last follow-up

N	KT 1000 side-to-side	Lysholm Score	Subjective IKDC	Objective IKDC	KOOS	Lachman test	Pivot Shift test	Howell grade
1	1 mm	97	94	A	97%	Grade 0	0	1
2	4 mm	96	95	B	98%	Grade I	1	2
3	1 mm	99	94	A	95%	Grade 0	0	1
4	2 mm	90	90	A	96%	Grade 0	1	2
5	2 mm	100	98	A	97%	Grade 0	1	1
6	3 mm	98	89	B	94%	Grade I	0	1

Lachman test was graded as suggested by Hurley et al. [26]: Grade 0, Tight with firm end-feel; Grade I, Nominal increase in laxity compared to contralateral knee; Grade II, Slight increase in anterior translation compared to contralateral knee; Grade III, Excessive anterior translation compared to contralateral knee.

Pivot shift test was graded as 0, absence; 1 + , glide; 2 + , clunk; 3 + , gross

**Fig. 2:**
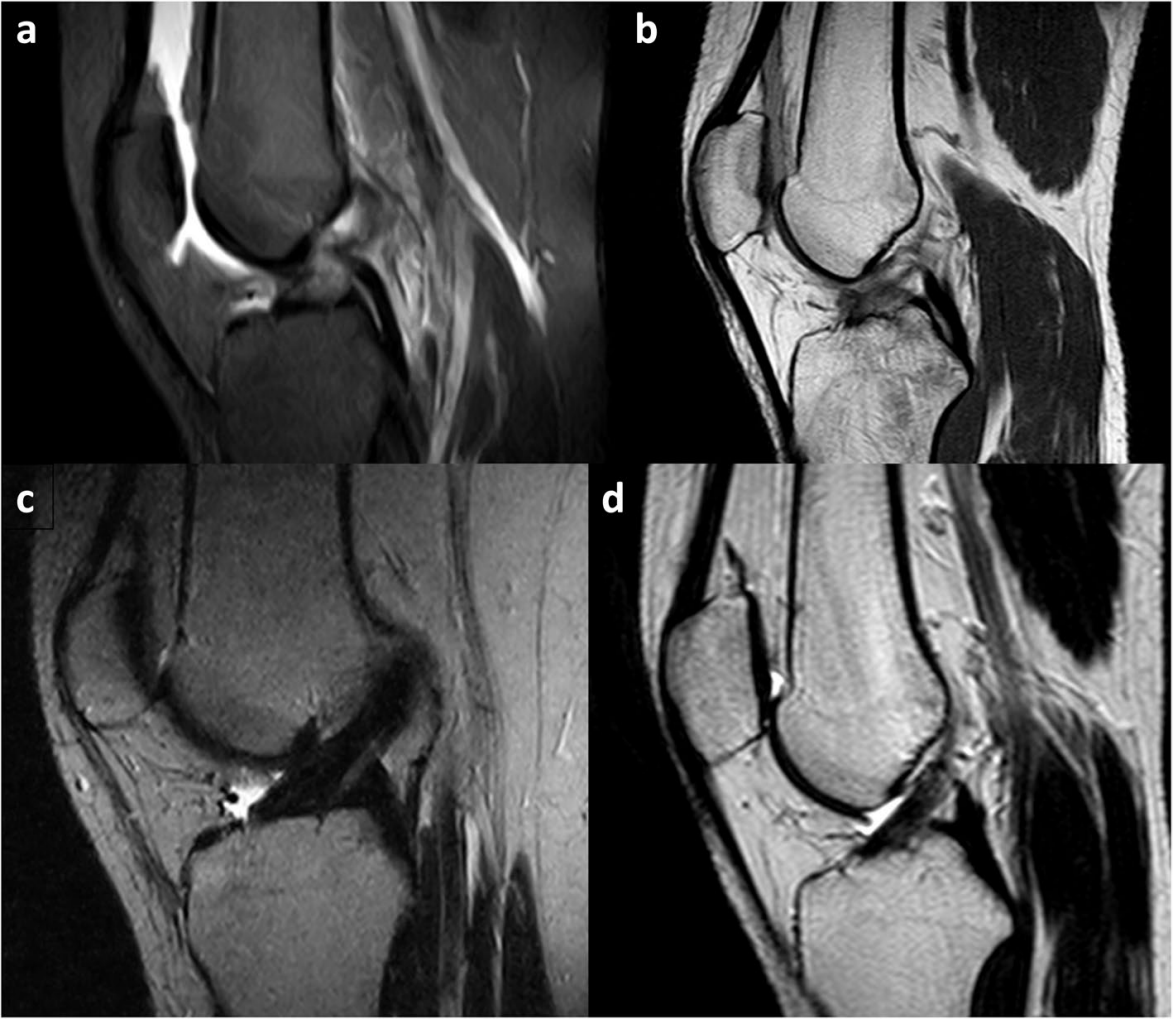
MRI appearance of patient 1 and patient 3 at the time of diagnosis and six months later, **a**, First MRI of patient 1 after knee injury in a short tau inversion recovery (STIR) sequence and, **b**, first MRI of patient 3 in a T2 weighted sequence, showing both swelling, complete discontinuity and increased signal of ACL. **c**, and **d**, MRI appearance in T2 weighted sequence of patients 1 and 3, respectively, showing complete recovery of signal intensity and continuity of anterior cruciate ligament.

In all patients, MRI at 6 months revealed an end-to-end continuous ACL with homogeneous signal and disappearance of the secondary signs (Fig. 2). The mean SNQ was 1.9 and the ligament was graded as ‘1’ for 5 patients and as ‘2’ for 1 patient according to Howell scale [27].

### Literature review

A search of comprehensive databases retrieved 1057 articles. After excluding 635 duplicates, 422 articles were screened. The screening and the selection of abstracts led to a total of 8 full-text articles that were assessed for eligibility according to the inclusion/exclusion criteria (Table 3).

**Table 3:** F, female; IKDC, International Knee Documentation Committee; LM, lateral meniscus; M, male; MCL, medial collateral ligament; MM, medial meniscus; MRI, magnetic resonance imaging; PCL, posterior cruciate ligament; PKT, physio kinesitherapy. * Seven patients who underwent the Khalifa treatment showed a continuous unsuspected ACL on MRI at follow-up. ** Data referred to the 15 patients of the Khalifa treatment group

**First Author (Year)**	**Type of study**	**No. Of Patients (Mean Age) Sex**	**Type of Lesion **	**Associated Lesions**	**Activity Level**	**Follow-up months (range)**	**Rehabilitation type (duration months)**	**Failure rate (%)**	**KT -1000, mm**	**IKDC**	**Lysholm score**	**Healing definition**
Ihara [28] (1994)	Prospective observational	25 + 7 PCL (23) 20 M / 12 F	/	24 LM 9 MM (6 LM + 1 MM for PCL lesions)	/	3	Kyuro knee brace (3) + Kinetec (0,5) + Dynamic joint control training (3) + Muscle strengthening exerces (3)+ No bearing (1) then partial bearing (0,5)	0 (0%)	/	/	/	Arthroscopy
Kurosaka [15] (1998)	Case Series	2 (18,5) 2 M / 0 F	(1) 1/3 proximal (0) midsubstance (1) 1/3 distal	1 MCL 1 MCL + MM	Competitive college sport participation	30 (24 to 36)	Double hinged brace. Unspecific rehabilitation protocol. (3-5).	0 (0%)	0,5 (range, 0-1)	/	/	Subjective Knee Function + Physical Examination + Arthroscopy
Malanga [16] (2001)	Case Report	1 (45) F	1/3 proximal	MCL	Dance instructor	19	No brace. No rehabilitation	0 (0%)	/	/	/	Physical Examination + Arthroscopy + RMI
Fujimoto [12] (2002)	Case Series	31 (33) 10 M / 21 F	/	0 meniscal lesions. No mention about other ligament.	Low athletic demand and sedentary occupation	16,1 (6 to 36)	Soft brace with 20-deg flexion block was applied for 3 months after the injury. Full weight-bearing withoutthe use of crutches was generally achieved within 4 weeks after the trauma. Jogging was started 5 months after surgery. (5)	8/31 (26%)	4	/	/	Subjective Knee Function + Physical Examination + MRI
Costa-Paz [11] (2012)	Case Series	14 (31) 12 M / 2 F	(8) 1/3 proximal; (6) mid-substance; (0) 1/3 distal	3 MM1 LL5 MCL	Recreational sport participation	25 (25 to 36)	No brace. Unspecific rehabilitationprotocol ( / )	2 had a reruptures (14%)1 had a meniscectomy (7%)	1,95 (Range 1-3,5)	10 Normal 4 Near normal	97 (range 90–100)	Subjective Knee Function + Physical Examination + MRI
Ofner [17] (2014)	Randomized Controlled Trial	30 (29,5) 14 M / 16 F	/	/	Recreational sport participation	• 1 group: Manual Khalifa Therapy + unspecific rehabilitation protocol (1,5) • 1 group: unspecific rehabilitation protocol (1,5)	23/30 failures (76%) *	< 2 (Max 5mm) **	89,27 (SD 10,5) **	89,27 (SD 10,5) **	/	Subjective Knee Function + Physical Examination + MRI
Jacobi [7](2016)	Jacobi [7](2016)	Jacobi [7](2016)	/	0 ligament lesion No data about meniscal lesions	/	24	• 1 group: ACL-jack brace with range of motion 0-100° (4) then FKT •. 1 group: No brace + PKT (2-4)	• 18/84 (21%) 13 failures + 5 reruptures • 6/20 (30%) failure	••••1,2 (± 2) 4,8 (± 2,5)	••••90 (± 8,7) 86.4 (± 11)	••••93.3 (± 8.3) 92.7 (± 7.4)	Subjective Knee Function + Physical Examination + MRI
Razi [10] (2021)	Case Series	15 (32) 8 M / 7 F	(15) 1/3 proximal (0) midsubstance (0) 1/3 distal	15 MCL (100%)2 Meniscus (13%)	Recreational sport participation	12	Brace for pain control + PKT ( / )	0 (0%)	/	/	/	Physical Examination + MRI

**Fig. 3:**
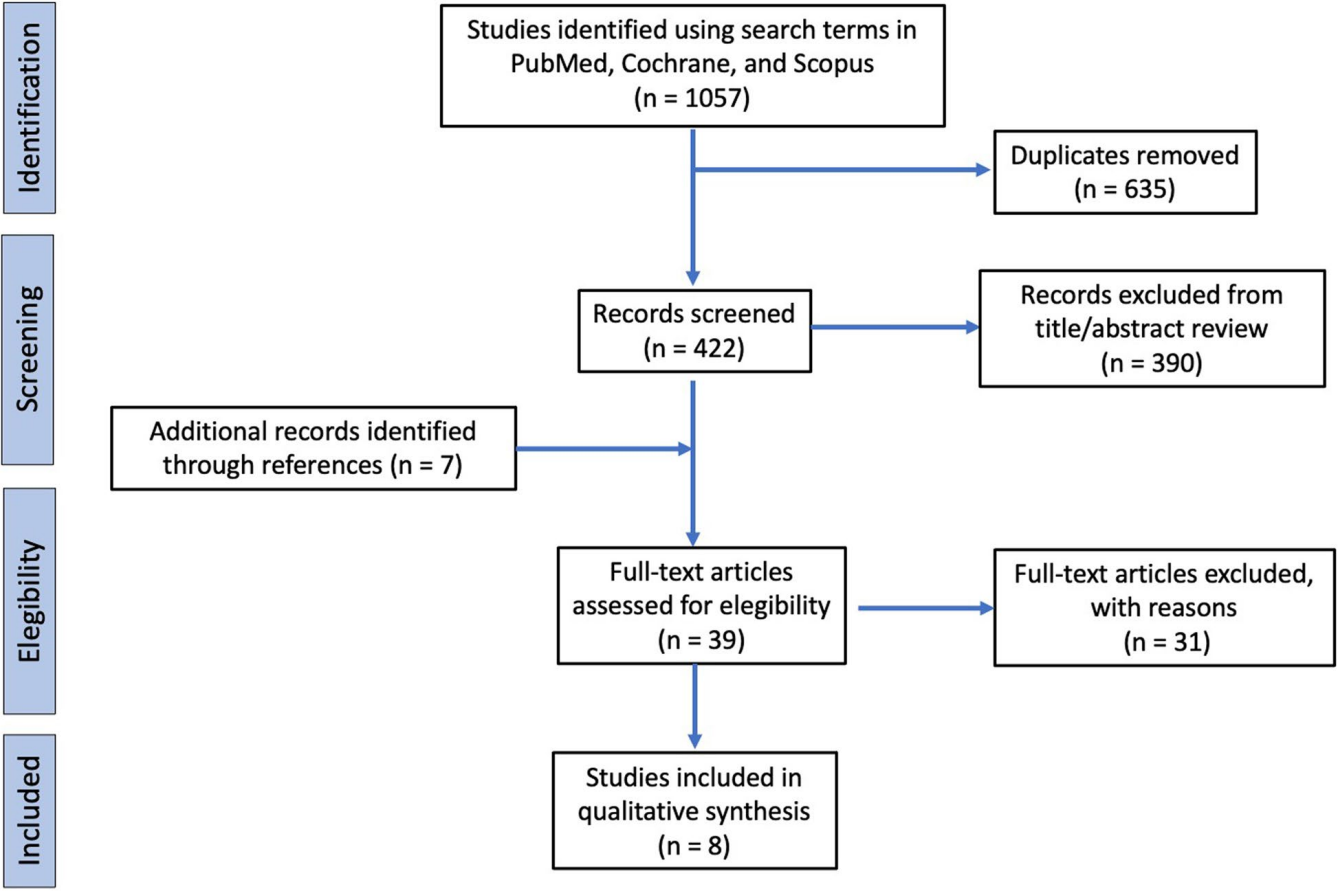
PRISMA (Preferred Reporting Items for Systematic Reviews and Meta-Analyses) flow diagram of the paper selection for literature review

The paper selection flow is represented in Fig. 3.

In 6 studies the patients underwent an MRI investigation at both post-injury and last follow-up, while in 2 studies patients underwent a diagnostic arthroscopy within 15 days of the injury and at the last follow-up. Six out of 8 studies included patients that underwent MRI at the time of injury and during the follow-up. The 2 papers that did not evaluate the ACL status by MRI were those by Kurosawa et al., in which the patients underwent a diagnostic arthroscopy within 8 days of injury, and Ihara et al., who performed a diagnostic knee arthroscopy within 15 days (range 2–15); in both the studies, an arthroscopic second look was taken at the end of follow-up. In the papers by Malanga et al. and Razi et al., the time from injury to MRI was not specified, but it can be deduced that was conducted within the third week after the trauma. Costa-Paz et al. did not specify the time at which the first MRI was carried out. In the other studies, the initial MRI assessment was performed within the first 4 weeks after injury.

The knee stability was clinically evaluated in all studies by Lachman test, KT-1000, and KT-2000 used as outcome measure tools. However, not all authors reported quantitative data in their results.

### Clinical outcomes

The studies were heterogeneous concerning the populations analysed regarding sport activity level, the treatment applied (different techniques and their combination, duration, and application modalities), and the evaluation methods (healing definition, follow-up).

The failure rate in the considered studies ranged from 0 to 73%, for a total of 50 patients. In Kurosaka et al.’s case report both patients returned to sport, but just for one of them was it specified that the pre-injury activity level was reached. The patient in Malanga et al.’s case report was only able to return to sport with a brace. All 14 patients in Costa-Paz et al.’s study returned to their pre-injury activity level. All 15 patients in Razi et al.’s paper returned to their sport, but it was not specified if they had any limitation. In other studies, data about the activity level achieved by patients after injury were not reported.

## Discussion

The main finding of the current paper was that the spontaneous healing of a ruptured ACL is possible, and the restoration of good knee function is achievable.

The patients reported in this article, both those in our case series and those in the other papers, received very heterogeneous treatments. Several authors treated their patients with a flexion brace, others with an extension brace, and others without a brace. Physical therapy was not always performed, and there is no consistency in the type of physical or manual treatment in the different papers included. The fact that is noticeable, however, when analysing the different papers is the distribution of ACL healing according to the site of injury. In the 4 papers included in the review that specified the ACL lesion site, spontaneous healing occurred in 25 cases with a proximal lesion, 6 cases with a middle substance lesion, and 1 case of distal rupture. In the current case series, all the patients experienced a proximal ACL tear: 4 patients sustained a type I ACL lesion and 2 patients had a type II injury, according to Van der list et al.’s radiographic classification of location of injury [22]. Those findings suggest that proximal-third lesions have higher healing potential. As reported in in vitro studies on vascularization of the ACL, the greater concentration of vessels and greater relative blood flow to the proximal third of the ACL suggest that this region may have greater healing potential. However, vascularity is not the only factor influencing the healing competence of a ruptured ACL. Some authors sustain that the lower capacity of the ACL to heal is related to specific intra-articular features and molecular biological characteristics [25]. Others have focused on the lower response of the ACL to growth factors compared to other ligaments [29]. Since evidence shows that the ACL has valid vascularization, especially in the proximal third [18, 30, 31], and the platelet clot seems able to deliver growth factor in an adequate manner to guarantee healing [19], the authors of this review believe that a lack of healing of a ruptured ACL is mainly anatomical or mechanical rather than biological [32]; abnormal tibial subluxation and reduced mechanotransduction can play their role.

Some authors have suggested that the use of a brace can prevent tibial anterior subluxation and any other displacement of the healing ACL. Prolonged use of a knee brace, especially if flexed, however, leads to severe stiffness after its removal and thus requires long rehabilitation periods to regain knee extension, with no certainty that it will lead to healing of the ACL. Ihara and Kawano [14] in 2017 conducted a study investigating the clinical and MRI outcomes of ACL-injured knees treated by a custom-made flexed knee brace to minimize abnormal sagittal translation movements between the femur and tibia. They reported the results for 102 patients, but the inclusion criteria were not clear on the ACL lesion type, they did not properly assess their definition of healing, and did not state how many patients failed this treatment. However, they reported an overall improvement after the treatment in anteroposterior translation measured by KT-1000 and ACL morphology and signal at MRI. The above-mentioned article by Ihara and Kawano was included in a recent systematic review conducted by Pitsillides et al. [33]. The authors believe that this systematic review has critical issues that differentiate it from the current paper, and this can be inferred from the selection of articles included in the review. Pitsillides and co-authors included and incorrectly reported the healing rate of the Ihara and Kawano paper (n = 7, which was not stated by the authors of the article themselves). Also, they included the article by Ihara from 1996 [13] that in the current paper was excluded because the definition of healing and failure rate were unclear. Still, unlike in the systematic review conducted by Pitsillides and co-authors, in the current paper the Ahn and Van Meer studies [34, 35] were excluded due to the lack of differentiation between total and subtotal ACL tears. Then, in their review the definition of healing was only based on MRI findings. The morphology and signal of the ACL on MRI can be misleading since it is common, during delayed ACL reconstruction surgeries, to observe that the proximal remnant of the torn ACL is sometimes attached to the surrounding tissues, such as the posterior cruciate ligament (PCL) or the intercondylar notch [19, 36]. This does not allow restoration of the original knee biomechanics and most of the time results in instability; the authors of the current study believe that clinical examination and a lack of giving-way symptoms should be the mainstay of the definition of healing. Finally, the populations, treatments, and outcomes (starting with the definition of healing) give too much heterogeneity to the patients among different articles included in the review. The authors of the current paper do not believe it is methodologically correct to pool their data and so do not consider Pitsillides et al.’s study design appropriate.

The current study has several limitations. Firstly, only 6 patients are reported from our database, which prevents any conclusion about non-surgical treatment of ACL lesions. Patients included both in the case series and in the review are heterogenous regarding age and activity level. Still, there is not a unique rehabilitation protocol, and healing was defined differently in the different papers. The functional results and return to sport were not clearly stated, the healing time was not specified, and no data were collected about risk factors. The authors believe that the greatest limit of the current study is the absence of epidemiological data about the incidence of spontaneous healing; moreover, the small number of patients precludes any analysis of predictive factors that would help us determine which patients might heal. Lastly, since only 3 databases were screened, it is possible that some studies have been missed in other databases. While this study highlights the emerging need for answers about non-surgically treated ACL outcomes, no conclusion can be drawn because of the low quality of the studies analysed and the heterogeneity of results.

Despite the study limitations, the authors of the review believe that a conservative period before reconstruction, in patients with low physical demand, might reduce the total number of surgeries performed with all the benefits this reduction might encompass, including reduced surgical risk for the patients and healthcare costs, and can lead to acceptable functional outcome.

## Conclusions

In conclusion, this review suggests that the ACL has healing potential and may heal under some conditions. Conservative treatment can lead to a successful recovery after an acute rupture; however, it is a rare event, and the authors remain convinced that surgical treatment (reconstruction or repair) is mandatory for athletic patients and strongly recommended for all active patients.

## Data Availability

The datasets generated during and/or analyzed during the current study are not publicly available but are available from the corresponding author on reasonable request.

## Abbreviations

ACL: Anterior cruciate ligament
F: Female
IKDC: International Knee Documentation Committee
LM: Lateral meniscus
M: Male
MCL: Medial collateral ligament
MM: Medial meniscus
MRI: Magnetic resonance imaging
PCL: Posterior cruciate ligament
PKT: Physio kinesitherapy
